# The nitric oxide‐cyclic guanosine monophosphate pathway inhibits the bladder ATP release in response to a physiological or pathological stimulus

**DOI:** 10.14814/phy2.14938

**Published:** 2021-07-20

**Authors:** Eriko Okuyama, Masahito Kawatani, Junichi Hashimoto, Keisuke Tanimoto, Manabu Hashimoto, Kazumasa Matsumoto‐Miyai

**Affiliations:** ^1^ Department of Radiology Akita University Graduate School of Medicine Akita Japan; ^2^ Department of Neurophysiology Akita University Graduate School of Medicine Akita Japan; ^3^ Graduate School of Comprehensive Rehabilitation Osaka Prefecture University Habikino Osaka Japan

**Keywords:** ATP release, LPS, NO‐cGMP, PDE5 inhibitor, urinary bladder

## Abstract

The release of ATP from the epithelium of the urinary bladder (urothelium) in response to mechanical/chemical stimuli contributes to the visceral sensation in the micturition reflex. The nitric oxide (NO)‐mediated induction of cyclic guanosine monophosphate (cGMP) has been detected in urothelial cells and may inhibit the micturition reflex. However, the function of the NO‐cGMP pathway in the regulation of urothelial ATP release remains poorly understood in contrast to its effects on smooth muscles or primary afferent nerves. Therefore, we investigated the relevance of the NO‐cGMP pathway to ATP release on the mucosal side in the present study. The administration of l‐arginine (NO precursor) or NOC 12 (NO donor) significantly reduced ATP release to the mucosal side at a physiologically normal urine storage pressure (5 cmH_2_O). L‐NAME (NO synthase inhibitor) significantly increased the distention‐induced release of ATP. The phosphodiesterase‐5 inhibitor, sildenafil, which increases cGMP levels, inhibited distention‐induced ATP release. Furthermore, sildenafil significantly reduced ATP release in response to the administration of lipopolysaccharide. These results suggest that the NO‐cGMP pathway inhibited urothelial ATP release during the storage phase under both physiological and pathological conditions.

## Graphical abstract

The release of ATP from the epithelium of the urinary bladder (urothelium) contributes to the visceral sensation in the micturition reflex. We showed that nitric oxide‐cyclic guanosine monophosphate (NO‐cGMP) pathway inhibited the bladder ATP release in response to distention or lipopolysaccharide. Our study suggested that the NO‐cGMP pathway suppressed the micturition reflex during the storage phase by reducing urothelial ATP release under physiological and pathological conditions.

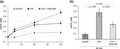

## 1 INTRODUCTION

The epithelium of the urinary bladder (urothelium) is regarded as a barrier that prevents the penetration of chemical agents in urine into the urinary bladder wall. In the past decade, the urothelium, which contains chemical, mechanosensory, and thermal sensors, was found to function as a visceral sensor. ATP is released from the urothelium during urine storage (Janssen et al., 2017; Winder et al., 2014) and transmits visceral sensory signals to the central nervous system via P2X_2/3_ at afferent nerve terminals in close proximity to the urothelium (Cockayne et al., 2000, 2005; Vlaskovska et al., 2001), and then controls the micturition reflex (Apodaca, 2004; Janssen et al., 2017; Winder et al., 2014). ATP release is also involved in hyperesthesia under pathological conditions. Abnormalities in ATP release and purinergic receptor expression have been implicated in interstitial cystitis or overactive bladder, which leads to frequent urination and bladder pain (Sun and Chai, 2004, 2006; Sun et al., 2001).

The gaseous molecule nitric oxide (NO) is an essential signaling compound involved in various cellular processes (Förstermann & Sessa, 2012). NO is produced from l‐arginine, which is catalyzed by nitric oxide synthase (NOS). NO is released as a neurotransmitter or paracrine factor, penetrates the plasma membrane, and activates NO‐sensitive guanylyl cyclase in nearby cells. Guanylyl cyclase produces cyclic guanosine monophosphate (cGMP). cGMP, in turn, activates cGMP‐dependent protein kinase (PKG). The NO‐cGMP pathway is well known for its endothelium‐derived signal for vascular smooth muscle relaxation. Another function was recently revealed; NO in the epithelium of the gastrointestinal tract functions in peripheral sensory transmission (Lies et al., 2014; Page et al., 2009).

In the bladder, the presence of NOS, guanylate cyclase, cGMP, and phosphodiesterase‐5 (PDE5) was confirmed or implied in the urothelium and Kahal interstitial cells (ICC cells) (Gillespie et al., 2004, 2005, 2006; Rahnama'i et al., 2015, 2013). Bladder NO plays a role in the micturition reflex. The intravesical administration of a NO donor increased the intercontraction interval (Ozawa et al., 1999). Furthermore, a NO scavenger induced bladder overactivity (Pandita et al., 2000), and cGMP protein kinase type I‐deficient mice showed an impaired relaxant response to nerve‐derived NO and consequent bladder overactivity (Persson et al., 2000). In humans, PDE5 inhibitors, which increase cGMP levels, effectively attenuated lower urinary tract symptoms (LUTS) (McVary et al., 2007; Porst et al., 2011, 2013). Collectively, these findings suggest that NO facilitates urinary storage by relaxing smooth muscle and/or inhibiting afferent nerve firing. However, the regulatory effects of the NO‐cGMP pathway on urothelial ATP remain unclear. Therefore, we herein examined the functional roles of the NO‐cGMP pathway on distention‐evoked ATP release to the mucosal side of the urinary bladder in order to clarify its contribution to urine storage under physiological conditions. In addition, we investigated whether the control of the NO‐cGMP pathway affects urothelial ATP release under pathological inflammatory conditions. The release of large amounts of urothelial ATP was induced by the administration of lipopolysaccharide (LPS) (Beckel et al., 2015; Silberfeld et al., 2020; Takezawa et al., 2016), and LPS‐evoked rapid bladder hyperactivity was mediated by P2X_2_ and P2X_3_ receptors (Takezawa et al., 2016). It is of interest to clarify whether the pharmacological modulation of the NO‐cGMP pathway inhibits LPS‐induced pathological ATP release.

## 2 MATERIALS AND METHODS

### 2.1 Animals

Six‐ to 10‐week‐old C57BL/6J male mice were used in the present study. All protocols performed in this study were approved by The Animal Research Committee of Akita University and Osaka Prefecture University, and followed the guidelines of the American Physiological Society for Animal Research.

### 2.2 ATP release assay using the Ussing chamber

ATP release to the mucosal side was examined as described previously by Matsumoto‐Miyai et al. (2011, 2016, 2012) and Takezawa et al. (2016). In brief, the vertically opened urinary bladder was mounted between the two halves of a customized Ussing chamber. The diameter of the circular hole between the chambers was 3 mm. Chambers were filled with Krebs solution (117 mM NaCl, 5.9 mM KCl, 2.5 mM CaCl_2_, 1.2 mM MgCl_2_, 24.8 mM NaHCO_3_, 1.2 mM NaH_2_PO_4_, and 11.1 mM glucose) with 95% O_2_/5% CO_2_ bubbling. Since the source of NO or cGMP might not be mucosa, we adopted the full‐thickness of bladder.

As a physiological mechanical stimulation, we applied hydrostatic pressure at 5 cmH_2_O to the serosal (smooth muscle) side for 40 min. Hydrostatic pressure at 5 cmH_2_O reflects the physiological range of pressure during urine storage. We focused the effect of the pressure not inducing the micturition reflex (5 cmH_2_O) on the ATP release than that of the pressure closer to the micturition threshold (15–20 cmH_2_O), because the excess of ATP release during the storage phase in response to such a low pressure would result in visceral hypersensitivity and frequent urination.

In another series of experiments with a pathological chemical stimulation, we administered bacterial LPS from *Escherichia coli* O111:B4 (final concentration of 0.5 mg/ml in Krebs solution) to the mucosal side with exposure for 40 min. The administration of LPS was previously shown to induce inflammatory changes in the bladder wall (Hoshino et al., 1999) and ATP release to the mucosal side (Beckel et al., 2015; Silberfeld et al., 2020; Takezawa et al., 2016).

In both experiments, 50 µl of Krebs solution was sampled from the mucosal side before and 5, 20, and 40 min after the initiation of stimuli. All chemical reagents were administered to the mucosal side of chamber 30 min before the initiation of stimuli. ATP levels in 50 μl of Krebs solution on the mucosal side of the chamber were assayed using the luciferin‐luciferase method (Kikkoman Co., Ltd.) according to the manufacturer's protocol. Standard lines were constructed in each experiment using 3 × 10^−7^, 3 × 10^−8^, 3 × 10^−9^, and 3 × 10^−10^ M ATP. Changes in ATP levels by stimuli were calculated by subtracting the value obtained before the initiation of these stimuli. To check the interferences of maximal dose of each chemical reagent in this study on luciferin‐luciferase reaction, we measured and compared the relative light units resulting from luciferin‐luciferase assays using standard ATP solutions with each chemical reagent or a corresponding vehicle. As shown in Supplementary Material, almost no significant interference of each reagent on luciferin‐luciferase reactions was confirmed.

The sample sizes in the results were distinct animal numbers, which means that each animal had just one run of experimentation.

### 2.3 Chemical reagents

The chemical reagents used in the present study were obtained from the following sources: l‐arginine from WAKO Pure Chemical Industries Ltd.; 1‐hydroxy‐2‐oxo‐3‐(N‐ethyl‐2‐aminoethyl)‐3‐ethyl‐1‐triazene (NOC 12) from Dojindo; NG‐nitro‐l‐arginine methyl ester (L‐NAME) from Cayman Chemical; sildenafil citrate from Tocris Bioscience; 1‐(5‐chlornaphthalene‐1‐sulfonyl)‐1H‐hexahydro‐1,4‐diazepine (ML‐9), and LPS from *E*. *coli* O111:B4 (purified by ion‐exchange chromatography) from Sigma‐Aldrich.

### 2.4 Statistical analysis

The significance of differences was examined using an unpaired *t*‐test. All data were expressed as the mean ± standard error of the mean.

## 3 RESULTS

### 3.1 NO inhibited distention‐induced ATP release to the mucosal side

**FIGURE 1:**
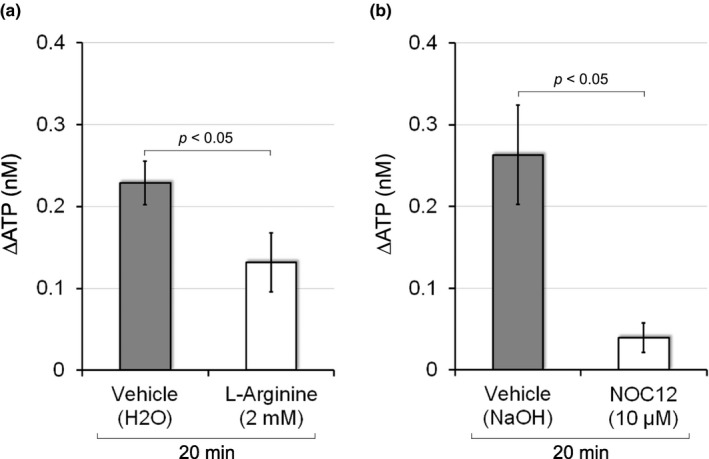
Effects of l‐arginine or NOC 12 on distention‐induced ATP release. (a) The effects of l‐arginine (2 mM) on physiological distention‐induced ATP release to the mucosal side of the chamber. Physiological distention of the bladder wall was induced by 5 cmH_2_O of hydrostatic pressure. The *Y*‐axis shows changes in ATP levels 20 min after distention. l‐arginine significantly inhibited ATP release elicited by distention of the bladder wall (*p* < 0.05 vs. vehicle by the unpaired *t*‐test). Error bars indicate SEM. Sample numbers are 28 for vehicle and 14 for l‐arginine. (b) The effects of NOC 12 (10 μM), a NO donor, on physiological distention‐induced ATP release to the mucosal side. The administration of NOC12 significantly reduced ATP release (*p* < 0.05 with the unpaired *t*‐test) in comparison to vehicle (0.1 N NaOH) 20 min after distention. Error bars indicate SEM. Sample numbers are 14 for vehicle and 8 for NOC 12

We initially investigated the effects of NO on ATP release to the mucosal side in response to the distention of the bladder wall by physiological pressure during urine storage (5 cmH_2_O). The NO precursor l‐arginine or NO donor NOC 12 was used to increase NO levels. The administration of l‐arginine (2 mM) significantly inhibited ATP release 20 min after distention (0.13 ± 0.04 nM with l‐arginine vs. 0.23 ± 0.03 nM with vehicle; *p* < 0.05; Figure 1). NOC 12 (10 μM) also significantly decreased ATP release 20 min after distention to approximately 15% (0.04 ± 0.02 nM with NOC 12 vs. 0.26 ± 0.06 nM with vehicle; *p* < 0.05; Figure 1). These results indicated that an increase in NO reduced distention‐induced ATP release.

### 3.2 Distention‐induced ATP release to the mucosal side was inhibited by endogenous NO

**FIGURE 2:**
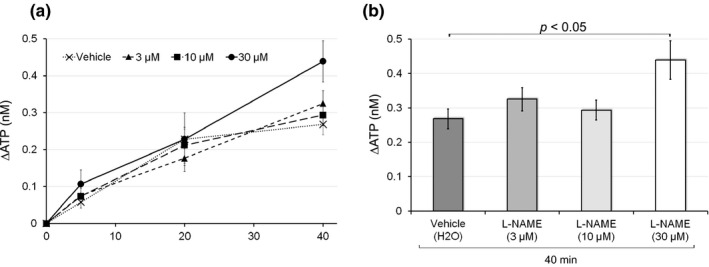
Effects of L‐NAME on distention‐induced ATP release. (a) The effects of L‐NAME (3, 10, or 30 μM) on physiological distention‐induced ATP release to the mucosal side. Line graphs show temporal changes in ATP release under each dose condition. The *Y*‐axis shows changes in ATP levels in the mucosal side of the chamber, which were measured 5, 20, and 40 min after the initiation of physiological pressure. Sample numbers are 28 for vehicle, 12 for L‐NAME (3 μM), 11 for L‐NAME (10 μM), and 5 for L‐NAME (30 μM). Error bars indicate SEM. (b) Bar graphs show the effects of different doses of L‐NAME on ATP release 40 min after physiological distention. A preincubation with L‐NAME (30 μM) significantly increased ATP release to the mucosal side (*p* < 0.05 vs. vehicle with the unpaired *t*‐test). Error bars indicate SEM

We then examined the effects of L‐NAME, a NO synthase inhibitor to assess the contribution of endogenous NO to distention‐induced ATP release. We investigated the effects of a preincubation with L‐NAME (3, 10, or 30 μM) on ATP release to the mucosal side 5–40 min after the initiation of physiological pressure (Figure 2a). Although low concentrations (3 or 10 μM) of L‐NAME had no effect, 30 μM of L‐NAME significantly enhanced ATP release 40 min after distention (0.44 ± 0.06 nM with L‐NAME [30 μM] vs. 0.23 ± 0.03 nM with vehicle; *p* < 0.05; 40 min after distention; Figure 2b). These results provide novel evidence to show that endogenous NO inhibits ATP release to the mucosal side at the late stage of urine storage.

### 3.3 cGMP inhibited distention‐induced ATP release to the mucosal side

**FIGURE 3:**
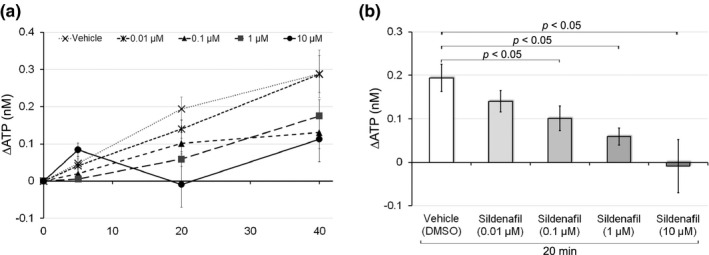
Effects of sildenafil on distention‐induced ATP release. (a) The effects of sildenafil (0.01, 0.1, 1, or 10 μM) on physiological distention‐induced ATP release to the mucosal side. Line graphs show temporal changes in ATP release under each dose condition. The *Y*‐axis shows changes in ATP levels in the mucosal side of the chamber, which were measured 5, 20, and 40 min after the initiation of physiological pressure. Error bars indicate SEM. Sample numbers are 15 for vehicle, 11 for 0.01 μM, 10 for 0.1 μM, 7 for 1 μM, and 11 for 10 μM of sildenafil. (b) Dose‐dependent effects of sildenafil (0.01, 0.1, 1, or 10 μM) on physiological pressure‐induced ATP release to the mucosal side 20 min after the initiation of physiological pressure. The administration of sildenafil reduced distention‐induced ATP release in a dose‐dependent manner. Significant differences were observed between the vehicle group and more than 0.1 μM groups (*p* < 0.05 with the unpaired *t*‐test). Reduction rates in the 0.1, 1, and 10 μM groups were 47.3, 69.5, and 104.7%, respectively. ATP release was almost completely abolished by 10 μM of sildenafil. Error bars indicate SEM

We investigated the effects of cGMP on distention‐induced ATP release to the mucosal side. cGMP is a downstream effector of the NO signaling pathway. Sildenafil, a PDE5 inhibitor, was used to increase cGMP concentrations. After a preincubation with sildenafil (0.01, 0.1, 1, or 10 μM), we examined ATP release to the mucosal side in response to physiological pressure (Figure 3a). Sildenafil gradually suppressed ATP release from 5 to 40 min. Twenty minutes after distention, 0.1, 1, and 10 μM of sildenafil significantly reduced ATP release in a concentration‐dependent manner (0.10 ± 0.03 nM with 0.1 μM of sildenafil vs. 0.19 ± 0.03 nM with vehicle, 0.06 ± 0.02 nM with 1 μM of sildenafil vs. with vehicle, −0.09 ± 0.06 nM with 10 μM of sildenafil vs. with vehicle; *p* < 0.05; Figure 3b). Physiological pressure‐induced ATP release was almost completely abolished by 10 μM of sildenafil. The inhibitory effects of sildenafil suggested that NO reduced distention‐induced ATP release via the generation of cGMP.

### 3.4 Sildenafil prevented SOCE blockade‐induced increases in ATP release in response to distention

**FIGURE 4:**
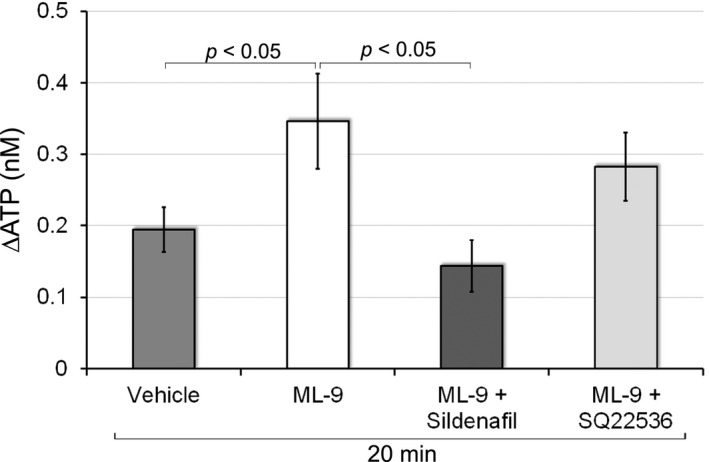
Effects of sildenafil or SQ22536 on the facilitation of ATP release by ML‐9. The *Y*‐axis shows changes in ATP levels in the mucosal side of the chamber 20 min after distention. ML‐9 (an inhibitor of SOCE; 100 μM) significantly enhanced urothelial ATP release by physiological distention (*p* < 0.05 vs. vehicle). The administration of sildenafil (10 μM) and ML‐9 abrogated ML‐9‐induced increases in ATP release (*p* < 0.05 vs. ML‐9), whereas the preincubation with SQ22536 (100 μM) did not affect the facilitatory effects of ML‐9. Error bars indicate SEM. Sample numbers are 15 for vehicle, 12 for ML‐9, 8 for ML‐9 + sildenafil, and 10 for ML‐9 + SQ22536. SOCE, store‐operated Ca^2+^ entry

We previously demonstrated that the blockade of store‐operated Ca^2+^ entry (SOCE) by ML‐9 facilitated distention‐induced ATP release to the mucosal side (Matsumoto‐Miyai et al., 2011). In the present study, we investigated whether the activation of the NO‐cGMP pathway inhibited ML‐9‐induced increases in ATP release. Consistent with our previous findings, ML‐9 significantly enhanced distention‐induced ATP release by approximately 178% (0.35 ± 0.07 nM with ML‐9 vs. 0.19 ± 0.03 nM with vehicle; *p* < 0.05; Figure 4). The administration of sildenafil and ML‐9 reduced ATP release to the mucosal side to a significantly lower level than that with the vehicle (0.14 ± 0.04 nM with ML‐9 + sildenafil; *p* < 0.05 vs. ML‐9; Figure 4). SOCE has been shown to activate NOS and inhibit adenylyl cyclase type 6 (Chiono et al., 1995; Fagan et al., 1998; Lin et al., 2000). Since our previous findings indicated that the adenylyl cyclase‐cAMP pathway enhanced ATP release, we also examined the effects of SQ22536 (an inhibitor of adenylyl cyclase). The administration of SQ22536 and ML‐9 slightly, but not significantly, decreased ML‐9‐induced increases in ATP release to the mucosal side (0.28 ± 0.05 nM with ML‐9 + SQ22536; Figure 4). This result indicated that the increase in cGMP by sildenafil also inhibited the chemically evoked excess of distention‐induced ATP release.

### 3.5 Sildenafil inhibited LPS‐evoked ATP release to the mucosal side

**FIGURE 5:**
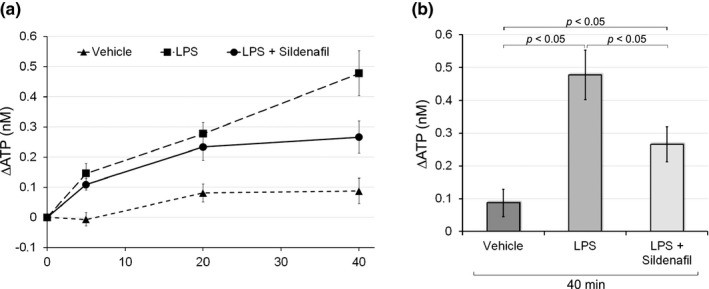
Effects of sildenafil on LPS‐induced ATP release to the mucosal side. (a) The effects of sildenafil (10 μM) on LPS (0.5 mg/ml)‐induced ATP release to the mucosal side. Line graphs show temporal changes in ATP release in response to the administration of vehicle, LPS alone, and LPS with a preincubation of sildenafil. The *Y*‐axis shows changes in ATP levels in the mucosal side of the chamber, which were measured 5, 20, and 40 min after the administration of LPS. Error bars indicate SEM. Sample numbers are 8 for vehicle, 13 for LPS, and 14 for LPS with a preincubation with sildenafil. (b) Bar graphs show the effects of different treatments on changes in ATP levels in the mucosal side of the chamber 40 min after the administration of vehicle, LPS alone, and LPS with a preincubation of sildenafil. The ATP level with the administration of LPS and sildenafil was still higher than that with vehicle (*p* < 0.05 vs. vehicle), but was significantly lower, by approximately 45%, than that with LPS alone (*p* < 0.05 vs. LPS). Error bars indicate SEM. LPS, lipopolysaccharide

We then examined the effects of sildenafil on ATP release to the mucosal side in response to the administration of LPS. A previous study demonstrated that the administration of LPS‐induced markedly higher levels of ATP release to the mucosal side of the urinary bladder than physiological pressure (5 cmH_2_O) (Takezawa et al., 2016). In the present study, similar results were obtained (Figure 5a); we confirmed that 0.5 mg/ml of LPS‐induced ATP release 40 min after its administration (0.48 ± 0.07 nM with LPS vs. 0.09 ± 0.04 nM with vehicle; *p* < 0.05 with the unpaired *t*‐test; Figure 5b), and the facilitatory effects of LPS on ATP release tended to be stronger than those of 5 cmH_2_O of hydrostatic pressure (Figures 2a, 3a, and 5a). Although a preincubation with sildenafil still induced a significant ATP release 40 min after LPS administration in comparison to vehicle control (0.09 ± 0.04 nM with vehicle vs. 0.27 ± 0.05 nM with LPS + sildenafil; *p* < 0.05; Figure 5b), sildenafil significantly decreased LPS‐induced ATP release (0.27 ± 0.05 nM with LPS + sildenafil vs. 0.48 ± 0.07 nM with LPS; *p* < 0.05; Figure 5b). This result indicated that the increase in cGMP by sildenafil inhibited pathologically evoked ATP release.

## 4 DISCUSSION

In the present study, we revealed that the NO‐cGMP pathway abrogated ATP release to the mucosal side of the urinary bladder in response to physiological pressure during urine storage (Figures 1, 2, 3). The effects of the NOS inhibitor L‐NAME and PDE5 antagonist sildenafil suggest that the NO‐cGMP pathway acts as an endogenous inhibitory regulator of ATP release. Whereas the removal of the urothelium had no effect on the release of NO (Munoz et al., 2010), NO release was observed in cultured urothelial cells (Birder et al., 1998, 2002). Furthermore, the expression of all NOS isoforms, namely, endothelial NOS (eNOS), neuronal NOS, and inducible NOS, in the urothelium has been reported (Birder et al., 2002; Satake et al., 2017). A previous study detected cGMP in the urothelium, suburothelial interstitial cells, and endothelium of blood vessels after the inhibition of PDE5 (Rahnama'i et al., 2013). Another study confirmed the inhibitory effects of sildenafil on ATP release using mucosa separated from the underlying detrusor (Chakrabarty et al., 2019). These findings indicate that the NO‐cGMP pathway is located in the urothelium or suburothelium.

Since urothelial ATP release triggers the visceral sensation in the micturition reflex, the inhibition of ATP release by the NO‐cGMP pathway may suppress the micturition reflex. The effects of L‐NAME on ATP release were only observed 40 min after the initiation of physiological pressure (Figure 2), which suggests that the NO‐cGMP pathway prolongs the urine storage phase by reducing ATP release in the late phase. The late increase observed in NOS activity might be due to the upregulation of NOS expression; shear stress on arteries, a mechanical stimulation as well as distention, induced eNOS expression 4 h after the stimulation (Woodman et al., 2005). Alternatively, the ATP metabolism might be affected by the NO‐cGMP pathway. The previous study speculated that one of the reasons why tamoxifen diminished the ATP content and increased the ADP content in rat liver might be due to the secondary inhibitory action of NO, whose production is stimulated by tamoxifen (Marek et al., 2011). In addition to the late effect, sildenafil reduced the rapid, transient increase in urothelial ATP release in response to brief distention (Chakrabarty et al., 2019), which may correspond to relaxation after spontaneous, low‐amplitude rhythmic bladder contractions without voiding. The NO‐cGMP pathway could inhibit ATP release induced by various mechanical stimuli during the entire storage phase.

The NO‐cGMP pathway has multiple functions in the urinary bladder, one of which was proposed to be relaxation of the smooth musculature. However, its contribution to smooth muscle relaxation in the lower urinary tract is still controversial; PDE5 inhibitors exerted relaxing effects on isolated surgical specimens of the urinary bladder, prostate, or urethra (Gacci et al., 2016), while other studies revealed the absence of cGMP responses in bladder smooth muscle (Gillespie et al., 2004; Lies et al., 2013). Another target of the NO‐cGMP pathway is the bladder afferent nerve; the nerve activities of Aδ and C fibers were decreased by l‐arginine or the PDE5 inhibitor tadalafil (Minagawa et al., 2012). This reduction in afferent nerve activities may be attributed to inhibitory effects on ATP release. Since the above‐described effects, namely, the relaxation of smooth muscle and reductions in afferent nerve activities and ATP release, result in the inhibition of the micturition reflex, the activation of the NO‐cGMP pathway may be an effective therapeutic strategy for storage dysfunctions in LUTS.

The regulation of the NO‐cGMP pathway is involved in bladder functions under chemically evoked pathological conditions. NO reductions by L‐NAME enhanced capsaicin‐induced detrusor overactivity (McVary et al., 2007). Furthermore, the NO substrate l‐arginine or PDE5 inhibitor tadalafil decreased the acrolein‐ or cyclophosphamide‐induced hyperactivity of bladder afferent nerves (Minagawa et al., 2012; Yu & Groat, 2013). The present results also showed that sildenafil significantly reduced the ML‐9‐induced excess of ATP release in response to distention (Figure 4). Since previous study showed that the sustained eNOS activity required the SOCE in COS‐7 cells (Lin et al., 2000), ML‐9 inhibiting SOCE would reduce cGMP content. Sildenafil might abrogate the ML‐9 effect on ATP release by inhibiting the degradation of the resultant small amount of cGMP. Previous studies revealed that *E*. *coli*‐derived LPS‐induced ATP release to the mucosal side (Beckel et al., 2015; Silberfeld et al., 2020; Takezawa et al., 2016) and rapid bladder hyperactivity in wild‐type mice (Takezawa et al., 2016). Bladder hyperactivity was attenuated in P2X_2_‐ or P2X_3_‐deficient mice (Takezawa et al., 2016), which suggested that ATP release caused bladder hyperactivity. We confirmed that LPS exerted an intense inductive effect on ATP release (Figure 5), and also revealed that sildenafil significantly decreased LPS‐evoked ATP release by approximately 45% (Figure 5b). The inhibitory effects of sildenafil on LPS‐evoked ATP release were weaker than those on distention‐induced ATP release because the latter was almost completely diminished by the same dose (10 µM) of sildenafil (Figure 3b). This partial inhibitory effect of sildenafil on LPS‐evoked ATP release may be due to the existence of two separate mechanisms for ATP release, pannexin channels and lysosomal exocytosis (Beckel et al., 2015; Silberfeld et al., 2020); LPS was previously shown to induce lysosomal exocytosis‐dependent ATP release (Beckel et al., 2015; Silberfeld et al., 2020), whereas ATP release in response to physical distention was mediated by pannexin channels and lysosomal exocytosis (Nakagomi et al., 2016). The present results indicate that both of the mechanisms underlying urothelial ATP release are differentially dependent on the NO‐cGMP pathway. The NO‐cGMP pathway was shown to abrogate both of ATP release mechanisms in a different way. NO inhibited the pannexin 1‐mediated currents in HEK‐293 cells through a cGMP‐PKG‐dependent pathway (Poornima et al., 2015). On the other hand, a nitrated derivative of cGMP, 8‐nitro‐cGMP, decreased the speed of individual spikes of exocytosis in bovine chromaffin cells, and this effect was independent of the PKG signal (Tsutsuki et al., 2020). The reason why the effect of sildenafil on lysosomal exocytosis of ATP induced by LPS was partial might be that the production of 8‐nitro‐cGMP is primarily dependent on the nitration reaction rather than the amount of cGMP. Another possible reason is that LPS activates a diversity of signaling pathways; LPS induces the release of multiple cytokines (Yeh et al., 2019), some of which may evoke NO insensitive ATP release. In any case, activation of the NO‐cGMP pathway could partially, but significantly attenuate bladder hyperactivity in cystitis.

In humans, the beneficial effects of PDE5 inhibitors have been anticipated in the treatment of LUTS. For example, PDE5 inhibitors improved IPSS scores for LUTS (Donatucci et al., 2011; McVary et al., 2007; Mulhall et al., 2006; Roehrborn et al., 2008; Sairam et al., 2002) and abnormal bladder symptoms in patients with spinal cord injury (Gacci et al., 2007; Taie et al., 2010). The present results support the possibility of extending drug indications for PDE5 inhibitors to storage symptoms accompanying visceral hypersensitivity.

However, our present study has some limitations including (1) the lack of confirmation that ATP released from the mucosal side of the bladder is really derived from the bladder epithelium, (2) the lack of measuring the stretch‐dependent ATP release at a higher bladder pressure (e.g., 15 cmH_2_O), which is still in the storage pressure range and could contribute more to storage LUTS, and (3) the lack of confirmation that the findings in *ex vivo* experiments are applicable to the control mechanism of in vivo bladder function. Further studies using the primary culture of urothelial cells, other physiological stimulus with a higher pressure, and *in vivo* analysis of voiding behavior will clarify the contribution of the NO‐cGMP pathway to the amelioration of storage symptoms.

In conclusion, we herein demonstrated the inhibitory effects of the NO‐cGMP pathway on bladder ATP release to the mucosal side in response to a physiological distention stimulus. The NO‐cGMP pathways were also involved in the significant reduction in ATP release in response to LPS stimulation occurring in the bacterial infection, even if the effect would be partial.

## DISCLOSURES

No conflicts of interest, financial or otherwise, are declared by the authors.

The authors acknowledge all funding sources supporting this work and all institutional or corporate affiliations. The authors certify that they had no commercial associations that may pose a conflict of interest in connection with the submitted article. The authors accept full responsibility for the conduct of this study, had full access to all of the data, and controlled the decision to publish.

## AUTHOR CONTRIBUTIONS

E.O., M.K., and K.M.‐M. conceptualized and designed the research; E.O., M.K., J.H., K.T., and K.M.‐M. performed the experiments; E.O., J.H., K.T., and K.M.‐M. analyzed the data; E.O., M.K., and K.M.‐M. interpreted the results of experiments; E.O., M.K., and K.M.‐M. prepared the figures; E.O., M.K., and K.M.‐M. drafted the manuscript; E.O., M.K., and K.M.‐M. edited and revised the manuscript; E.O., M.K., J.H., K.T., M.H., and K.M.‐M. approved the final version of the manuscript.

## Supporting information



Supplementary Material
