# Supplementary material for: The nitric oxide‐cyclic guanosine monophosphate pathway inhibits the bladder ATP release in response to a physiological or pathological stimulus
Source: Physiol Rep. 2021 Jul 20;9(14):e14938. doi: 10.14814/phy2.14938 (PMC8290832; doi:10.14814/phy2.14938)
Supplement: Supplementary file 1 — Supplementary Material [file PHY2-9-e14938-s001.pdf]

SUPPLEMENTAL DATA

The presence of chemical reagents did not significantly interfere with the luciferin-luciferase reactions.

To check the interferences of maximal dose of each chemical reagent in this study (10 μM of NOC 12, 2 mM of L-arginine, 30 μM of L-NAME, or 10 μM of sildenafil) on luciferin-luciferase reaction, we measured the relative light units resulting from luciferin-luciferase assays using standard ATP solutions with each chemical reagent or a corresponding vehicle (0.1 N NaOH for NOC 12, H_2O for L-arginine and L-NAME, or DMSO for sildenafil). We performed two independent experiments using 3×10^{-7} M (300 nM), 3×10^{-8} M (30 nM), 3×10^{-9} M (3 nM), and 3×10^{-10} M (0.3 nM) of ATP solutions under each condition. The average values of relative light units in two measurements were shown in Supplemental Table 1, and the graphs based on these values were shown in Supplemental Fig. 1. As indicated by these data, almost no significant interference of each reagent on luciferin-luciferase reactions were detected.

For Review Only

Supplemental Table 1. Relative light units in luciferin-luciferase assays with chemical reagents or the corresponding vehicles

Chemical Reagent	ATP Concentration (nM)			
	0.3	3	30	300
Vehicle (0.1 N NaOH)	30.5	306.5	3099.5	31722
NOC 12 (10 μM)	27.5	304	3136.5	30452
Vehicle (DMSO)	28.5	312.5	3102	31390
Sildenafil (10 μM)	29.5	312	3141.5	31593
Vehicle (H_2O)	28	300	3147.5	32227
L-arginine (2 mM)	31	303.5	3081	31754.5
L-NAME (30 μM)	30	308.5	3089.5	30444.5

For Review Only

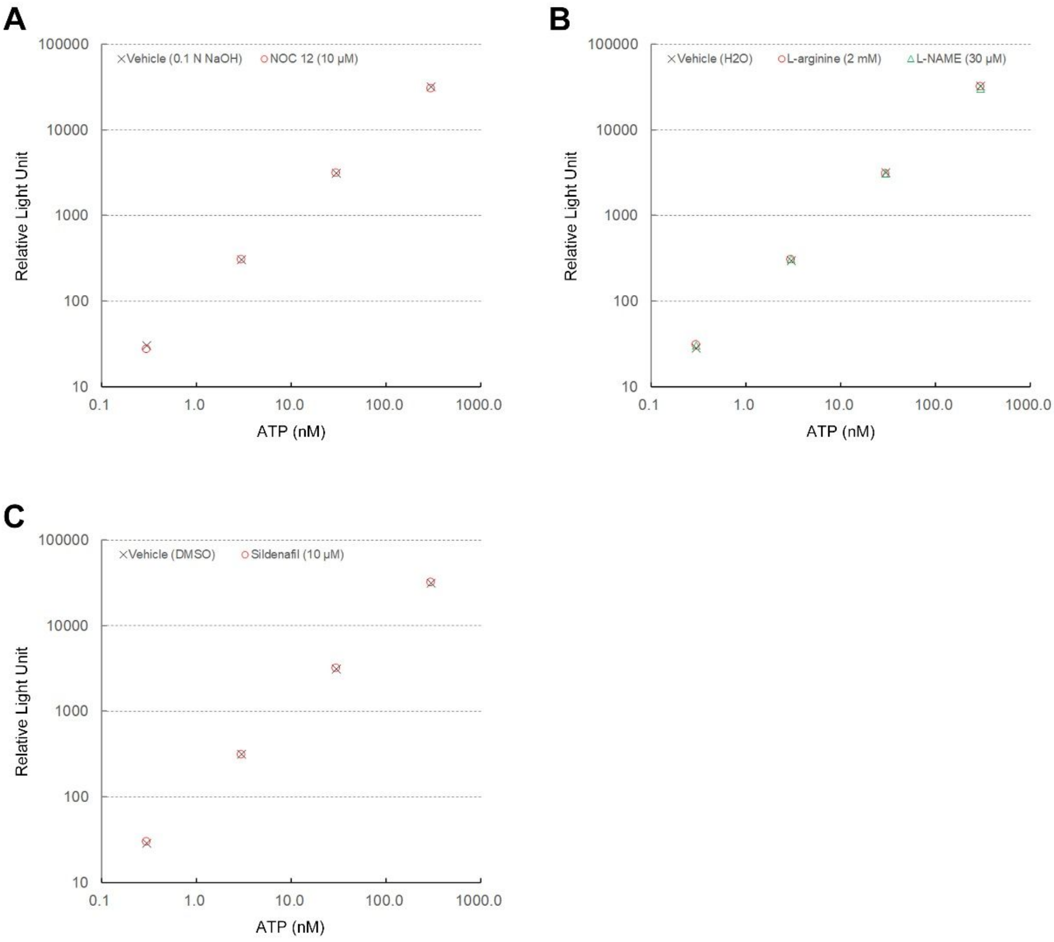

Supplemental Figure 1

Only

SUPPLEMENTAL FIGURE LEGENDS

Suppl. Fig. 1. Influences of chemical reagents on luciferin-luciferase reactions.

The effect of NOC 12 (**A**), L-arginine (**B**), L-NAME (**B**), sildenafil (**C**) on luciferin-luciferase reactions in comparison to the corresponding vehicles (**A**; 0.1 N NaOH, **B**; H_2O, **C**; DMSO). Each graph shows the relative light units (the Y-axis) in luciferin-luciferase reactions using the standard ATP solutions (0.3, 3, 30, or 300 nM; the X-axis). There was no significant difference between relative light units with chemical reagents and those with corresponding vehicles.

For Review Only
